# Double vertical interrupted suture for optimal adaptation and stabilization of free gingival graft around dental implants: a case report

**DOI:** 10.1186/s13256-024-04611-2

**Published:** 2024-06-26

**Authors:** Neda Moslemi, Amirmohammad Dolatabadi, Seyedhossein Mohseni Salehimonfared, Fatemeh Goudarzimoghaddam

**Affiliations:** 1 Department of Periodontics, School of Dentistry, Tehran University of Medical Sciences, Tehran, Iran; 2 Dental Research Institute, Dental Research Center, School of Dentistry, Tehran University of Medical Sciences, Tehran, Iran

**Keywords:** Suture, Dental implants, Free gingival graft, Autogenous grafts

## Abstract

**Background:**

Free gingival graft is commonly used to augment the keratinized mucosa and vestibular depth around dental implants. The proper suturing technique is fundamental to achieve a successful result following free gingival graft. However, there are limited studies that focus on the details of the suturing methods to optimize graft adaptation. The purpose of this technical note is to describe a new suturing technique for optimal approximation and stabilization of free gingival graft around dental implants.

**Case presentation:**

Here, we present a 53-year-old Persian female with peri-implantitis and lack of keratinized mucosa around mandibular implants who was a candidate for free gingival graft. A new suturing technique, double vertical interrupted suture, was conducted in the interimplant areas. In addition, the suspensory cross-mattress sutures were added to ensure the adaptation of the graft over the implants. The proposed suturing technique is useful for soft tissue augmentation around multiple implants with concave or uneven recipient bed.

**Conclusion:**

The present article describes a novel suturing technique for good adaptation and fixation of free gingival graft around dental implants.

## Background

One of the most crucial elements for long-term peri-implant stability is establishing sufficient supra-crestal soft tissue seal and an adequate band of keratinized attached tissue. This soft tissue barrier protects the underlying bone from microbial invasion and subsequent peri-implant diseases [1]. Over the past five decades, free gingival graft (FGG) has been widely used as the most documented and predictable procedure [2, 3] to increase the width of keratinized tissue and vestibular depth around the implants [4–6]. FGG is not free of complications, including bleeding, tooth sensitivity, ecchymosis, and graft necrosis owing to suture loosening [7]. Moreover, full/partial necrosis and dimensional shrinkage following FGG have remained a long-standing and unanswered problem, especially around dental implants [8–11]. This shrinkage most often occurs in terms of width compared with the length [12].

Several factors have been identified to be responsible for undesirable outcomes of FGG. Among them, improper preparation of the recipient bed and inadequate adaptation and fixation of the graft are paramount [6]. The lack of stability and adaptation of the graft over the recipient area jeopardizes different stages of the healing process, including plasmatic circulation and revascularization [13, 14]. In addition, adequate approximation and fixation of the FGG into the recipient bed is most often difficult to achieve, especially in sites with high muscle pull and limited vestibular depth, in the presence of scarred periosteum or uneven recipient bed. Some techniques have been described to stabilize the FGG, including suturing techniques [15, 16], titanium tacks [13], acrylic stent [17], and cyanoacrylates [18, 19].

In a randomized clinical trial by Shammas *et al*., horizontal continuous and apical stretching sutures were compared with interrupted sutures regarding postoperative shrinkage of FGG around the single tooth. They found no significant difference between the two groups regarding graft shrinkage following surgery. The lack of difference in the investigated methods was associated with the occurrence of tissue trauma owing to an increased number of needle insertions, which increased the possibility of graft shrinkage [16].

Liao *et al*. introduced a periosteal suturing technique for dental implants to secure the FGG over the recipient bed in the coronal borders of the graft. The authors stated this suturing technique might be impossible in cases with thin phenotype tissues [15].

Recently, Abdallah *et al*. proposed using titanium tacks instead of sutures for FGG fixation. Reduced operation time has been reported as the main advantage of this fixation method. However, the authors recommended avoiding the use of this technique in areas with a history of guided bone regeneration or previous local infection since there may not be adequate cortical bone thickness to stabilize the tacks [13].

Although both cyanoacrylate [18, 19] and customized acrylic/composite surgical stent [17] are considered as effective alternatives for FGG fixation, one of the limitations associated with these alternative methods is that clinicians may encounter challenges regarding the accessibility to the necessary materials and resources. Furthermore, customized surgical stents are associated with an increased cost and additional chairside time to fabricate the stent.

Routinely, the clinicians use a number of single interrupted sutures to fix the FGG to the surrounding intact attached tissue or the nearby periosteum. Although this method is useful for graft fixation, adequate and thorough approximation of the graft to the underlying tissue is often not achieved through such a suturing technique. On the other hand, numerous perforations of the graft through several interrupted sutures might induce trauma to the graft and increase the risk of graft necrosis and scar formation [20].

Some clinicians suggested an additional cross mattress sling suture, anchored to the healing abutment, to compress the graft against the bed in the central portion of the implant. Whereas this suturing technique is beneficial for graft adaptation over the implant site, such suture techniques could not achieve graft adaptation in areas between the implants. This is especially true when the implants are distant from each other [11, 15].

In the present technical note, we have introduced a novel suturing method for optimal adaptation and stabilization of the FGG around dental implants, with the least number of sutures penetrating the graft.

## Case presentation

**Fig. 1:**
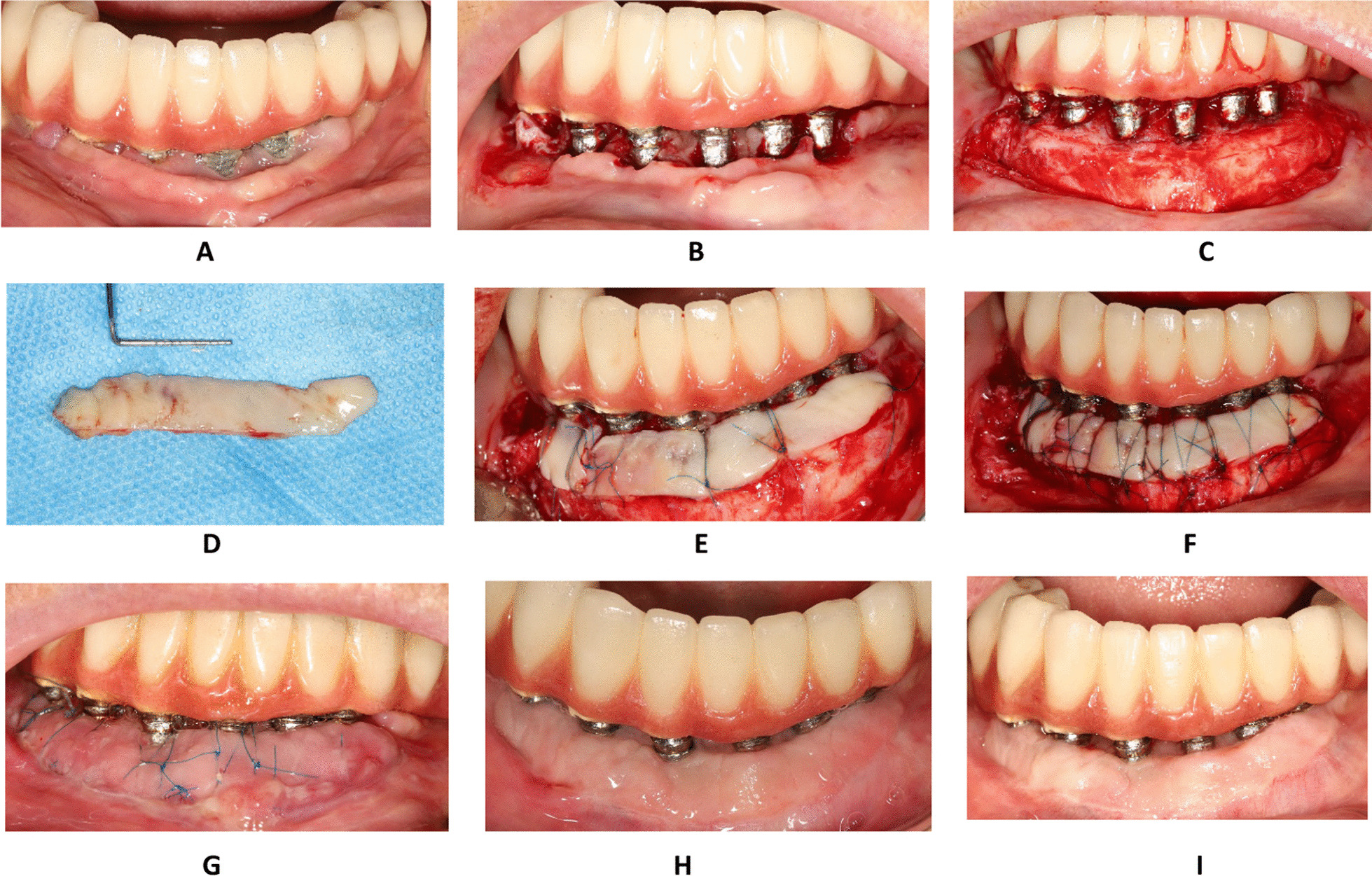
Free gingival graft in lower mandible of a patient with peri-implantitis. **A** Baseline. **B** The exposed threads were removed by using implantoplasty burs, and then the surfaces of the implants were polished and disinfected. **C** Recipient bed. **D** The graft was harvested from palate. **E** Double vertical interrupted suture was used to optimize graft adaptation in between the implants. **F** Additional suspensory cross-mattress sutures were used over each implant. **G** The 2-week follow-up. **H** The 2-month follow-up visit. **I** The 6-month follow-up

A 53-year-old Persian female, who is systemic-healthy and a nonsmoker, complained of pain and discomfort around the implants during tooth brushing, was used to illustrate this technique. The patient had received a full-mouth implant-supported fixed prosthesis 2 years ago. During clinical examination, two to three fixture thread exposure was observed on most mandibular implants. The maximum probing depth was 3 mm. Using rolling probe test [21], lack of keratinized mucosa and vestibular depth deficiency around dental implants was found. According to the clinical and radiographic examinations, peri-implantitis was diagnosed. The etiology of peri-implantitis may be attributed to a combination of factors, such as inadequate oral hygiene, insufficient keratinized mucosa, and over-contoured and nonmaintainable prosthesis (Fig. 1A, B). Therefore, FGG and implantoplasty were planned to reconstruct the lack of keratinized mucosa and vestibular depth, eliminate the plaque retentive surfaces, and enhance oral hygiene care. Before the surgical intervention, the patient underwent nonsurgical peri-implant therapy and was carefully monitored for 1 month to ensure significant improvement in her oral hygiene. The surgical procedures were performed by one experienced practitioner in the private clinic (NM). Following verbal and written informed consent, the patient was enrolled in this study. For this case, the recipient bed was prepared as follows: After delivery of local long buccal/block anesthesia, a horizontal supraperiosteal incision was made about 15 mm far from the mucosal margin in the desired vestibular depth and was extended 5 mm beyond the distal surface of the most distal implants. This long horizontal incision is directed coronally at the distal ends. Then, a coronal, horizontal supra-periosteal incision was performed at the level of the mucosal margins of the implants, traversing the mucosal margins of the adjacent implants, and joining the apical incision in the most distal parts of the bed. Subsequently, the entire unattached supraperiosteal soft tissue inside the incision lines (including mucosa, submucosa, and part of underlying muscles) was removed and the underlying periosteum remained on the prepared bed (Fig. 1C). The epithelialized FGG was harvested from the hard palate using a 15c blade (Swann-Morton, Sheffield, England). The graft thickness was about 1.5 mm. The fatty tissues were removed from the inner part of the graft (Fig. 1D). The graft was placed over the recipient site and the initial fixation was achieved by two single interrupted sutures in the most mesial/distal-coronal parts of the graft, which engaged the adjacent intact attached tissues.

### Suture technique description

**Fig. 2:**
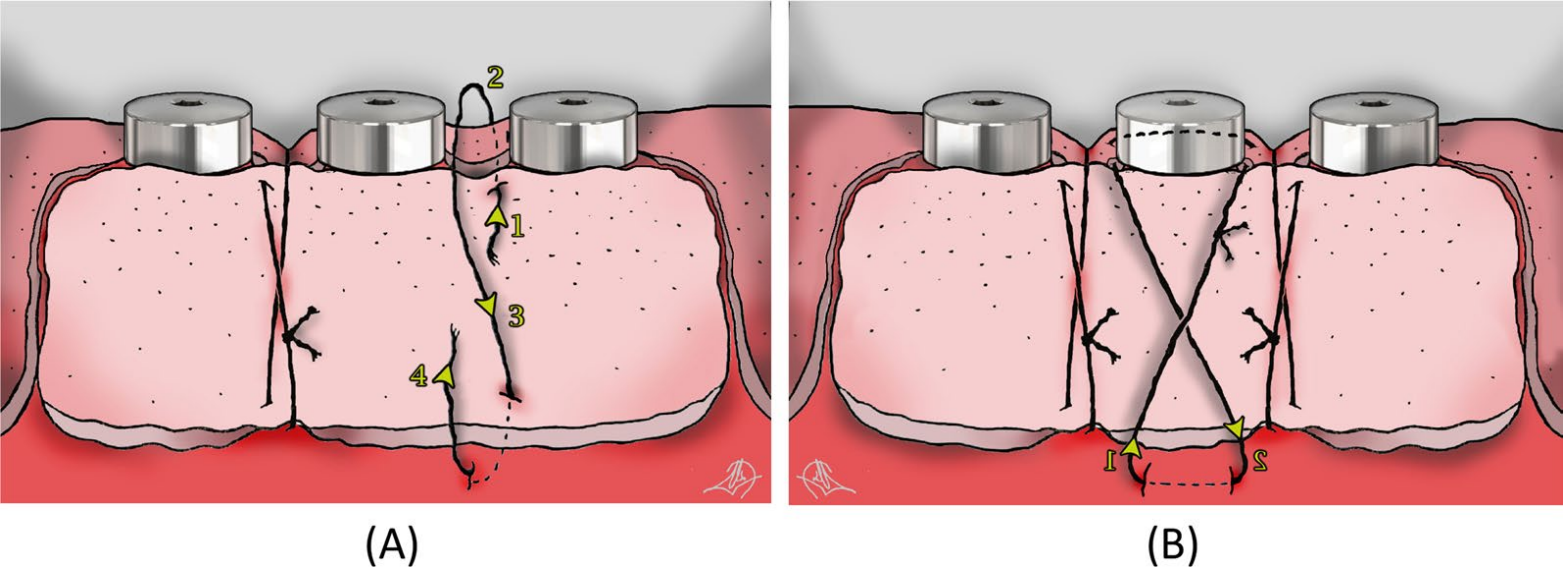
Schematic view of suturing sequences for free gingival graft around multiple dental implants. **A** Double vertical interrupted suture is conducted in the interimplant areas. **B** The suspensory cross-mattress suture is applied to ensure the adaptation of the graft over each implant

Double vertical interrupted sutures were conducted in the inter-implant areas and the mesial/distal ends of the graft as follows: first, the needle of the suture (6.0 or 7.0, needle length: 11–14, Nylon) entered the coronal aspect of the graft, approximately 2 mm from the graft margin, engaging the underlying attached mucosa or underlying periosteum passed through the lingual/palatal tissue of the bed and existed at the palatal/lingual tissue. Then, the needle was brought back to the buccal aspect and entered the graft approximately 2 mm from the apical margin, along with the starting point of the needle in the coronal aspect. The needle engaged the underlying periosteum apical to the graft. Subsequently, a surgeon’s knot was used to secure the suture (Figs. 1E and 2A). Significantly, any tension or compression might have jeopardized the blood supply of the graft during the healing phases and had to be avoided. Finally, additional suspensory cross-mattress sutures were used over each implant to optimize the vascular supply from the underlying bed to the graft over the implants (Figs. 1F and 2B).

### Suture removal and clinical outcome

At 2 weeks following an uneventful healing, the sutures were removed (Fig. 1G). The patient had minimal postoperative discomfort and morbidity. FGG with a double vertical interrupted suturing technique resulted in successful results in terms of keratinized mucosal width and vestibular depth (Fig. 1H and I). In addition, the patient reported high satisfaction with the treatment outcome.

## Discussion

Among various surgical techniques described as “periodontal/peri-implant plastic surgeries,” FGG is most vulnerable to ischemia and avascular necrosis. Unlike pedicle grafts, survival of FGG is solely dependent on the blood supply derived from the underlying bed. Therefore, maintaining the approximation of the graft to the underlying bed to prevent the blood clot from growing in this interface is crucial to achieve a successful outcome. This is particularly the case with dental implants, compared with natural teeth. The less predictability of the soft tissue augmentation procedure around dental implants has been attributed to the reduced vascularity of peri-implant soft tissue compared with that of periodontal tissue [22, 23].

In addition, the more apical the graft position, the more challenging to achieve an optimal adaptation and stabilization of the graft. Physiologic ridge resorption following tooth extraction leads to implant placement and subsequently the FGGs in a more apical position and closer to the vestibular depth, where the graft fixation will be more difficult to achieve. Another issue is the risk of periosteal rupture during bed preparation and graft fixation, which makes satisfactory stabilization of the graft impossible. The previous procedure for implant placement and probably flap advancement might impair the structure of the periosteum to a less elastic tissue. Compared with the intact periosteum, the scarred periosteum is more prone to rupture, especially following several manipulations for engaging the apical periosteum during nearby single interrupted sutures. Therefore, satisfactory graft fixation in the apical region is often difficult or impossible to accomplish.

The advantages of using double vertical interrupted sutures are: (1) to optimize the stability of the graft in the coronal and apical directions simultaneously and (2) to approximate the graft to the underlying bed with the least number of sutures. This type of suture is beneficial in areas between the implants and is most useful in cases with multiple implants far from one another. Supplementary suspensory cross-mattress sutures are suggested for graft adaptation over the buccal aspect of the implants. Most often, using the combination of these two types of sutures will be enough to attain fixation and adaptation of the FGG around dental implants and additional trauma caused by multiple insertions of the needle into the graft and underlying periosteum will be avoided. To facilitate periosteal suturing and prevent periosteal rupture, we recommend avoiding administration of subperiosteal infiltration anesthesia, and instead we usually use long buccal or block anesthesia. The local infiltration subperiosteal anesthesia will elevate the periosteum from the underlying bone and increase the risk of periosteal rupture during suturing.

Conventional FGG bed preparation consists of apically repositioning mucosa. However, we propose removing the unattached supra-periosteal soft tissue inside the incision lines instead of apically repositioning the mucosa. In this manner, the structures that interfere with the graft fixation are eliminated, and the visibility and accessibility of the surgeon for suturing the graft to the apical periosteum are highly enhanced.

Connective tissue graft (CTG) is also considered as an important autogenous graft for peri-implant soft tissue augmentation [6]. CTG is usually combined with coronally advanced flap [24–26] or tunnel [27] technique to increase soft tissue thickness or height. The fixation and adaptation of CTG are equally important as those of FGG for similar reasons. Therefore, this suturing technique is recommended for the fixation of CTG to the underlying periosteum. Whereas some suturing techniques have been described for improving the success of tunnel technique or coronally advanced flap [28, 29] around dental implants, to our knowledge, there are limited studies focused on individual suturing techniques for fixation of FGG around dental implants [15].

Compared with the recently reported technique [16] double vertical interrupted suture is associated with fewer numbers of needle insertions, which results in less trauma to the graft. In addition, our suturing technique can be used in cases with thin phenotype, opposed to the recently proposed technique [15].

Using tacks for graft fixation has been proposed to reduce the surgical time [13]. However, there are some concerns about using tacks instead of sutures: (1) using tacks is usually not applicable in sites with a lack of adequate thickness of the cortical bone, such as in areas that have undergone guided bone regeneration or previous local infection; (2) although tacks provide an excellent fixation of the graft, they are not able to guarantee a sufficient adaptation all through the graft over the recipient site, especially in cases with concave or uneven surfaces; and (3) usually, it is not possible to control the amount of compressive force on the graft during the insertion of the tack into the bone. Therefore, the risk of pressure necrosis of the graft following tack insertion needs to be considered.

## Conclusion

The double vertical interrupted suture can be useful for improving graft stability and adaptation following the FGG procedure. More studies are necessary to validate the effect of this suturing technique on clinical outcomes and to compare this technique with other conventional methods.

## Data Availability

Data will be available on request.

## Abbreviations

FGG: Free gingival graft
MGJ: Mucogingival junction
CTG: Connective tissue graft
